# SNP analysis of challenging bone DNA samples using the HID-Ion AmpliSeq™ Identity Panel: facts and artefacts

**DOI:** 10.1007/s00414-023-03019-9

**Published:** 2023-05-22

**Authors:** Paolo Fattorini, Carlo Previderè, Tommaso Livieri, Tomaž Zupanc, Irena Zupanič Pajnič

**Affiliations:** 1 Department of Medicine, Surgery and Health, University of Trieste, Trieste, Italy; 2 Department of Public Health, Experimental and Forensic Medicine, Section of Legal Medicine and Forensic Sciences, University of Pavia, Pavia, Italy; 3 Institute of Forensic Medicine, Faculty of Medicine, University of Ljubljana, Ljubljana, Slovenia

**Keywords:** SNPs, PCR-MPS, DNA degradation, LT-DNA

## Abstract

PCR-MPS is an emerging tool for the analysis of low-quality DNA samples. In this study, we used PCR-MPS to analyse 32 challenging bone DNA samples from three Second World War victims, which previously yielded no results in conventional STR PCR-CE typing. The Identity Panel was used with 27 cycles of PCR. Despite that we only had an average of 6.8 pg of degraded DNA as template, 30 out of 32 libraries (93.8%) produced sequencing data for about 63/90 autosomal markers per sample. Out of the 30 libraries, 14 (46.7%) yielded single source genetic profiles in agreement with the biological identity of the donor, whereas 12 cases (40.0%) resulted in SNP profiles that did not match or were mixed. The misleading outcomes for those 12 cases were likely due to hidden exogenous human contamination, as shown by the higher frequencies of allelic imbalance, unusual high frequencies of allelic drop-ins, high heterozygosity levels in the consensus profiles generated from challenging samples, and traces of amplified molecular products in four out of eight extraction negative controls. Even if the source and the time of the contamination were not identified, it is likely that it occurred along the multi-step bone processing workflow. Our results suggest that only positive identification by statistical tools (e.g. likelihood ratio) should be accepted as reliable; oppositely, the results leading to exclusion should be treated as inconclusive because of potential contamination issues. Finally, strategies are discussed for monitoring the workflow of extremely challenging bone samples in PCR-MPS experiments with an increased number of PCR cycles.

**Supplementary Information:**

The online version contains supplementary material available at 10.1007/s00414-023-03019-9.

## Introduction

In forensic genetics, the current gold standard for human identification is multiplex PCR of polymorphic markers followed by capillary electrophoresis (CE) separation of the amplicons [1, 2]. Whilst STR (short tandem repeats) are mainly analysed in routine analyses, SNPs (single nucleotide polymorphisms) and InDel (insertion/deletion) markers may be characterised in highly degraded samples [1–5].

Current PCR-CE technology is not sensitive enough to type some of the low copy number (LCN) and/or degraded DNAs [1, 2]. In such cases, even if several strategies have been deployed, the resulting genetic profile is often of scarce or even no utility [6, 7].

In the last decade, massive parallel sequencing (MPS) technology has been implemented in forensic laboratories [8], and several kits, which allow for the simultaneous multiplexed typing of hundreds of markers, have been developed and customised. In addition, the sensitivity of new technology has increased, as well [8–10]. Therefore, PCR-MPS offers several advantages over conventional PCR-CE analysis, in particular for the analysis of low template degraded samples [8–10].

SNP analysis of challenging samples has consistently yielded better results than STR typing [3, 4]. The simple molecular structure of SNP polymorphisms does not allow the production of stutters and other PCR artefacts usually found when STR markers are amplified [1, 5]. In addition, the reduced molecular size of the amplicons makes SNP markers the best choice in the genetic typing of degraded samples [4, 5].

One of the commercialised PCR-MPS kits is the HID-Ion AmpliSeq™ Identity Panel, which allows the simultaneous typing of 90 autosomal (plus 34 Y-specific) SNPs leading to combined random match probabilities (RMP) between 1×10^−34^ and 1×10^−37^ [11–23]. In the last few years, several studies have used this kit for typing low copy and/or degraded samples on ion torrent machines [24–28]. Although the analytical conditions (number of PCR cycles, concentration of the pooled libraries, and thresholds for locus call, etc.) and the sets of samples wildly differed, the results univocally highlighted the advantages of the PCR-MPS approach. In fact, all the studies concluded that the discrimination power (i.e. the RMP) remains high even if only low percentages of markers were successfully typed [24–28]. It is therefore possible that LCN degraded samples, which have previously yielded no results following the conventional STR PCR-CE approach, could be successfully typed by the employment of the HID-Ion AmpliSeq™ Identity Panel.

When employing samples with very low DNA yields or even undetectable DNA, increased number of PCR cycles seems to be the most promising approach for successful outcomes [1, 2, 6, 25, 27]. Since PCR reaction with high number of cycles is more prone to artefacts [1, 2], the aim of our study was to check the usefulness of the Identity Panel with 27 cycles of PCR on very challenging samples. In addition, because aged skeletal samples are prone to contamination, we also investigated whether the employment of SNP PCR-MPS technology with an increased number of cycles can lead to the detection of exogenous human DNA contamination that was not identified using standard STR PCR-CE technology.

Recently, we described the successful STR PCR-CE genetic typing of 112 out of 144 bone specimens sampled from different anatomical regions of three Second World War victims [29]. Since the remaining set of 32 samples yielded no STR typing results, the original DNA aliquots of those samples were used for PCR-MPS analyses of identity SNPs in this study. The results of the Precision ID Identity Panel typing, using 27 cycles of PCR in MPS technology, are shown and discussed.

## Materials and methods

### Ethic statement

The study was approved by the Ethical Committee of the Republic of Slovenia (KME 102/11/14).

### Samples

Our analyses included 32 challenging bone samples. Alongside these samples, an additional 15 samples (reference bone samples, positive PCR control, and low template degraded control samples) were analysed (Table S1).

#### Challenging samples

We selected thirty-two bone extracts whose STR PCR-CE typing gave no result when employing the Investigator® ESSplex SE QS (Qiagen) at 30 PCR cycles. The samples were originally extracted from three different male skeletons (skeleton A, B, and C). The remains are approximately 75 years old and were found in the mass grave Huma Jama (Slovenia), from the Second World War [29]. DNA quantification was carried out by the PowerQuant Kit at standard conditions. This kit provides reliable results down to 0.0001 ng DNA per μL of extract [30], whereas the IPC (Internal Positive Control) probe is able to detect the presence of PCR inhibitors. The challenging bone sample extracts showed detectable levels (> 0.0001 ng/μL) of DNA, with 24 out of 32 samples showing un-calculable degradation levels because of the lack of amplification of the 249 bp-long target. No inhibitors were detected [29].

#### Reference samples

Three to four bone samples for each skeleton were selected to generate the corresponding reference profile. The selection criteria were a high DNA yield and that a full PCR STR-CE profile was achieved in the original study [29]. These 11 samples showed Auto/Deg ratios between 4.1 and 14.6 (median value= 9.5) (Table S1).

#### Positive PCR control and low template degraded control samples

When dealing with challenging bone samples, the quality and quantity of the positive controls are of crucial importance. Since it is worthless to process 1 ng of DNA of high-molecular weight DNA for 27 PCR cycles as a positive control, we decided to analyse cellular and sub-cellular amounts (2, 5, and 10 pg) of the PCR positive control 2800M purchased from Promega (see Table S2). To check the performance of the PCR-MPS assay on low template (LT) degraded samples, diluted amounts (10 pg) of each of the skeletons’ reference samples were used for analysis. In addition, we used three other bone samples as LT degraded controls (one sample of 32 to 94 pg of DNA per skeleton) (Table S1). These bone samples were selected from our previous study because they gave successful STR PCR-CE typing [29]. Finally, we included one more sample as a LT degraded control. This sample was 10 pg of DNA from sample FM-24, an in vitro depurinated DNA sample already used in our previous PCR-MPS trial [31] (see Table S1).

#### Negative extraction controls

In our laboratory, we process an extraction negative control (ENC) in each batch of DNA extracted from bones; usually the batch contains 6 to 12 samples. In total, eight ENCs were processed alongside the 32 challenging bone samples; none of them provided amplicons in the STR PCR-CE experiments with 30 PCR cycles [29]. All eight ENCs were tested in the present study; however, to reduce the costs of the PCR-MPS reagents, three ENCs were randomly selected for the PCR-MPS analysis (Table S2). The remaining five ENCs were tested by STR PCR-CE analysis using an increased number of PCR cycles than what was suggested by the manufacturer (see paragraph “Assessing the identity of the samples”).

### Library construction, template preparation, and MPS sequencing

The Precision ID Identity Panel (TFS) was used for library construction and 90 autosomal SNP markers and 34 Y-specific SNPs were investigated. As shown in Table S2, 65 libraries were prepared according to the user guide [32]. One nanogram of DNA, as assessed by the Auto probe of the PowerQuant kit, was used for the analysis of the eleven reference samples at 24 cycles of PCR. Fifteen microliters (i.e. the maximum volume possible to use in the PCR reaction) was used for each of the challenging bone samples at 27 cycles of PCR. The DNA of the cell line 2800M was used as positive PCR control (PCR + ctrl) at the final dilution of 2, 5, and 10 pg. With duplication of each dilution, a total of six libraries were obtained from PCR + ctrl samples. LT degraded controls (LT deg ctrl) were represented by 10pg dilutions of four reference bone samples (one sample from skeleton A and skeleton B and two samples from skeleton C) and one artificially degraded sample (as for PCR + ctrl samples, analysis of FM-24 sample was duplicated).

Undiluted LT degraded controls (one bone sample of poor quality per each skeleton) were amplified using 32 pg of DNA (skeleton B), 51 pg of DNA (skeleton C), and 94 pg of DNA (skeleton A). Three ENCs and four NTCs (no template controls) were processed alongside the challenging samples. Twenty-seven PCR cycles were used for all control samples.

Fully automated library preparation was performed using the Precision ID Identity Panel and Precision ID DL8 Kit™ for Chef, and 32 libraries were combined into one tube for Ion 530™ chips following the manufacturer’s user guide [32]. The concentration of the combined library pool was determined by qPCR with the Ion Library TaqMan Quantification Kit™ (TFS) in duplicate together with standards and negative controls [33, 34]. Library pools (30 pM combining 32 samples) were used for fully automated DNA template preparation on the Ion Chef™ System. The templates were prepared using the Ion S5 Precision ID Chef™ Reagents and loaded using the single chip loading workflow. Sequencing made use of Ion S5™ Precision ID Sequencing Reagents and Ion S5™ Precision ID Sequencing Solutions.

### Sequencing data analysis and genotyping

The alignment of reads against the Homo sapiens reference genome (GRCh37/hg19) was performed using Ion Torrent™ Suit Software 5.10 (TFS) [33]. Coverage analysis was carried out with the Coverage Analysis v 5.6.0.1 plugin, which provides statistics and graphs describing the level of sequence coverage produced for targeted regions. Information about mapped reads, on-target percentage, mean depth, and uniformity of coverage were downloaded for each sample library (Barcode Summary Report file).

For genotyping, the Converge™ software version 2.0 (TFS) [35] was used by applying the manufacturer’s default settings. In particular, a minimum coverage of 20 × is required for genotyping, with each strand with more than 10 × of coverage. A MAF (major allele frequency) flag is assigned by the analysis software to heterozygous genotypes when the reads of the two alleles are unbalanced (10.1–35.0% or 65.0–89.1%), whereas homozygous genotypes are alerted when reads corresponding to a second allele account for 5 to 10% of the entire coverage of the marker.

### Definition of the reference profiles

The reference profile of skeletons A, B, and C was defined by using the genotyping data of three to four good-quality reference samples per skeleton. To this aim, the genotyping data were analysed by using the method recently described by Turchi et al. [27].

### PCR-MPS fidelity in challenging samples

For each sample, the number of markers above the threshold for locus call (e.g. 20 reads) was computed. In addition, since full consensus profile was yielded for each skeleton (see below), the following values were calculated for each PCR-MPS test of the challenging samples: number of autosomal allelic drop-out (ADO), number of allelic drop in (ADI), and number of Y-specific allelic drop in (and their frequencies). The frequency of MAF flags was calculated as well.

The same calculations were performed for the positive controls (PCR positive controls and LT degraded controls).

### Consensus profile from the challenging samples

The genotyping data of the challenging samples A, B, and C were used to build the *consensus* profile for the corresponding skeleton. To this aim, the method proposed by Turchi et al. [27] was used with minimal modifications.

### Assessing the identity of the samples

In order to assess if the challenging bone samples could be correctly assigned to the skeleton from which they were collected, the PCR-MPS autosomal genotyping data of each test was compared to the *reference* genotypes of the corresponding skeleton. To this aim, the LRMix software (version 2.1.5) [36] was used under the hypothesis that a single source DNA yielded the genotype. This tool, originally developed to calculate the LR (likelihood ratio) in mixed/low copy DNA samples analysed with STR systems, has been successfully employed even in the analysis of SNP markers [37]. The LRMix software is a semi-continuous interpretational model, which does not take in consideration the peak height information whereas allelic drop-out and allelic drop-in probabilities are evaluated. The following propositions were assumed in the present study: prosecution hypothesis (*PH*): the *reference* skeleton sample is the contributor of the tested bone sample; number of unknown contributors: 0; defence hypothesis (*DH*): the *reference* skeleton sample is not the contributor of the tested bone sample; number of unknown contributors: one. In addition, three different parameter settings were set up. According to the first one, the default settings were used (ADO frequency = 0.1; ADI frequency = 0.05). The second setting consisted in the ADO frequency as evaluated by the software by using the “*drop out estimation*” option (with a minimum of 0.1) whereas the ADI frequency was maintained at 0.05. The last setting stated that the ADO and ADI frequencies, as emerged from the analysis of each sample, were used. The *θ* correction value was fixed at 0.01 in all analyses. Caucasian allele frequencies, freely available at https://www.ncbi.nlm.nih.gov/snp/ (access: January 11, 2023), were used as reference database. This test was restricted to samples showing at least 19 typed markers, an arbitrarily threshold number fixed by us (see paragraph “LRMix analysis of challenging samples”).

The strength of the LR values of this test was verbally expressed in agreement with the ENFSI (European Network for Forensic Sciences Institutes) recommendation [38], with minimal modification. In particular, LR values from 0.5 to 2.0 were considered of “no support”, from 2.1 to 999 of “weak/moderate support”, from 1000 to 1,000,000 of “strong support”, and above 1,000,000 of “extremely strong support” to the prosecutor hypothesis, which is of the positive identification of the skeleton. The reciprocal values were expressed by the same verbal expressions in favour of the opposite hypothesis, which is the exclusion of the skeleton identification.

As six challenging samples gave likelihood ratio (LR) values < 1 when compared with the corresponding *reference* profile, the genotyping data of such samples were compared with the *reference* genotype of the other two skeletons. Thus, for example, the genotyping data of library #32 (which was built with sample C_11) were compared with the *reference* of skeleton A and skeleton B. In addition, in order to assess if positive LR values could emerge by chance when only a limited number of markers are typed, these six partial profiles were compared even with the genotypes of samples 2800M and FM, as well as with other ten unrelated individuals already studied with the Precision ID Identity Panel [27]. The corresponding LRs were calculated setting up the ADO frequency as evaluated by the “*drop out estimation*” option, and an ADI frequency of 0.05.

Finally, in order to calculate accurate LR values to understand whether a given skeleton can be the contributor of the challenging bone samples, even the quantitative continuous model EuroForMix [37, 39] was used. In this model, full peak height information (i.e. in the present paper, the number of reads) was considered together with allelic drop-outs and drop-ins frequencies for calculating the probability of obtaining a reference profile, given all possible genotype combinations of the contributors. The calculations were performed under the hypothesis of a single contributor to the evidence setting up the same analytical threshold considered by the Converge™ software for the allelic calls (20 reads). The quantitative LR model (maximum likelihood–based) was selected and the LR values were recorded after the sensitivity test.

### PCR-CE analysis of the ENCs

Five ENCs were submitted to analysis of STRs using increased number of cycles. As stated above in paragraph “Negative extraction controls”, these ENCs have yielded no amplicon when analysed through the employment of the Investigator® ESSplex SE QS (Qiagen) kit at the standard number of 30 cycles of PCR [40]. To further test these ENCs, 17.5 μL of each sample was amplified using the PowerPlex ESI 17 Fast (Promega) in a final volume of 25.0 μL by increasing the PCR cycles to 33. Standard procedures were used for CE analysis [1, 40].

### Calculations and graphs

Microsoft Excel 2007 and Stata/SE version 12.1 (StataCorp) were used for calculations and graphs. For statistical analyses (*t*-test, *χ*^2^, and ANOVA), significance was assumed with *p-*values *<* 0.05.

## Results and discussion

In this study, we analysed 32 challenging bone samples, 11 good-quality bone samples from three reference skeletons (A, B, C), and 22 controls by PCR-MPS using the Precision ID Identity Panel. The results achieved from the 65 libraries are described as follows.

### Libraries preparation

As reported elsewhere [29], qPCR quantification of the challenging samples yielded very low DNA concentrations with median and maximum values of 0.00045 and 0.0028 ng/μL, respectively. A further limitation was the maximum volume of DNA extract (15 μL) which can be used in PCR reaction of the Identity Panel kit. These data together suggested to set a suitable number of PCR cycles in order to have enough amplicons to prepare the libraries. There are very few papers that investigated the possibility of increasing the number of PCR cycles with this commercial kit. When only low amounts of template DNA are available, twenty-six cycles are recommended by the manufacturer [32], even if less cycles could be enough [25]. At the same time, however, user guide states that “cycle numbers can be increased if the quality or quantity of input DNA is uncertain” [32], and Turchi et al. showed that the employment of 31 cycles (23+8 cycles) leads to an improvement of the main sequencing parameters together with the number of markers with a suitable coverage [27]. Therefore, the extreme low amounts of template DNA available from the challenging Second World War bone samples led us to the decision to increase the number of PCR cycles to 27 for increasing the possibility of successfull library preparation. In addition, since all bone samples contained no more than 20 μL of DNA because the rest of DNA was used in our previous study [29], we preferred to carry out a single PCR test per sample by using the maximum available volume of 15 μL rather than split the samples into two PCR tubes to run duplicates. The knowledge of the genetic profile of the three skeletons (see below), in fact, allowed the evaluation of both the fidelity of the single PCR-MPS test and the assessment of the identity of the sequencing data, irrespective of the duplicates. The median DNA total amount for the PCR amplifications was of 6.8 pg, with a maximum of 42 pg. The concentration of the libraries, estimated by qPCR, always allowed sequencing template preparation at the recommended final concentration of 30 pM combining 32 samples [32, 33].

### DNA sequencing

Sixty-five libraries (11 from the reference samples, 32 from challenging samples, and 22 from control samples) were run in three different Ion 530™ chips. The chips also contained other samples that are not included in the present study. In total, 15 libraries were built from challenging samples of skeleton A, 6 from skeleton B, and 11 from skeleton C. The main sequencing parameters of the 65 libraries are shown in Figure S1.

The 32 challenging samples showed lower mapped reads, lower percentage of on-target reads, and lower mean of depth (*p*-value < 0.0006) than the positive controls (PCR + ctrl plus LT degraded controls). No difference was computed for the uniformity of the sequencing (*p*-value = 0.421). The results are in line with the sequencing of libraries built from very low amounts of degraded samples [12, 24, 25, 27].

The average values of the libraries of the reference samples were as follows: mapped reads = 729,859, percentage on-target reads = 97.4%, mean depth of sequencing = 5578, and uniformity = 97.5%; all values agree with the input of an optimal amount of template DNA (1 ng) [32].

### Autosomal typing

#### Threshold for locus call and negative controls

The use of a threshold for the “locus call” (e.g. its genotyping) has been debated from the very beginning of the PCR-MPS era in forensic genetics, and a minimum of 20 read has been proposed as a reliable threshold [12]. To date, a wide range of thresholds, from 6 × to 200 ×, have been used in different studies characterising the Precision ID Identity Panel [12–14, 23–28]. In addition, a comparison performed on three different thresholds (20, 50, and 100 ×) showed that genotyping errors occur even if the highest threshold is applied to the data [27]. In this study, we opted for the default setting threshold (20 reads) of the Converge™ software version 2.0 (TFS) because we were expecting low coverages from the challenging bone samples.

Another crucial point when performing PCR-based experiment is that blank controls (both ENC and NTC) should provide negative results [1, 2, 6, 41, 42]. As shown in Table S2, all four NTCs and two out of three ENCs showed only two markers with more than 20 reads of coverage. The third ENC (library #59), on the opposite, exhibited 12 markers above the threshold (with a median coverage of 773 reads), thus suggesting that a contamination issue occurred most likely during the DNA extraction procedure of the corresponding batch of samples. It is noteworthy that the analysis of the same ENC *via* PCR-CE did not yield any result after 30 cycles of PCR, as previously reported [29].

#### Reference profile of the three skeletons

Full *reference* autosomal SNP profiles of the three skeletons were generated through the analysis of optimal amounts (1 ng) of DNA and the comparison of the typing results. All the eleven samples used as reference samples, in fact, showed full profiles (90 out of 90 autosomal markers); in addition, the bone samples belonging to each specific skeleton yielded identical profiles. The average heterozygosity of the genotypes of the three skeletons was 0.411, 0.522, and 0.511 (A, B, and C, respectively), what is in agreement with the Hardy-Weinberg equilibrium (*p*-values > 0.236). The *reference* autosomal identity SNP profile is shown in Table S3 for skeleton A, Table S4 for skeleton B, and Table S5 for skeleton C (second column, highlighted in red).

#### PCR-MPS fidelity of the challenging samples

Out of the overall scored 2880 autosomal markers, 1899 were above the 20 × threshold in the 32 challenging samples, meaning that, in average, 65.9% of the markers were typed (51.6%, 80.2%, and 77.7% in skeletons A, B, and C, respectively; see Figure S2). The number of markers per sample ranged from 2 to 85, highlighting a huge sample-to-sample variability (see Figure S3A).

**Fig. 1:**
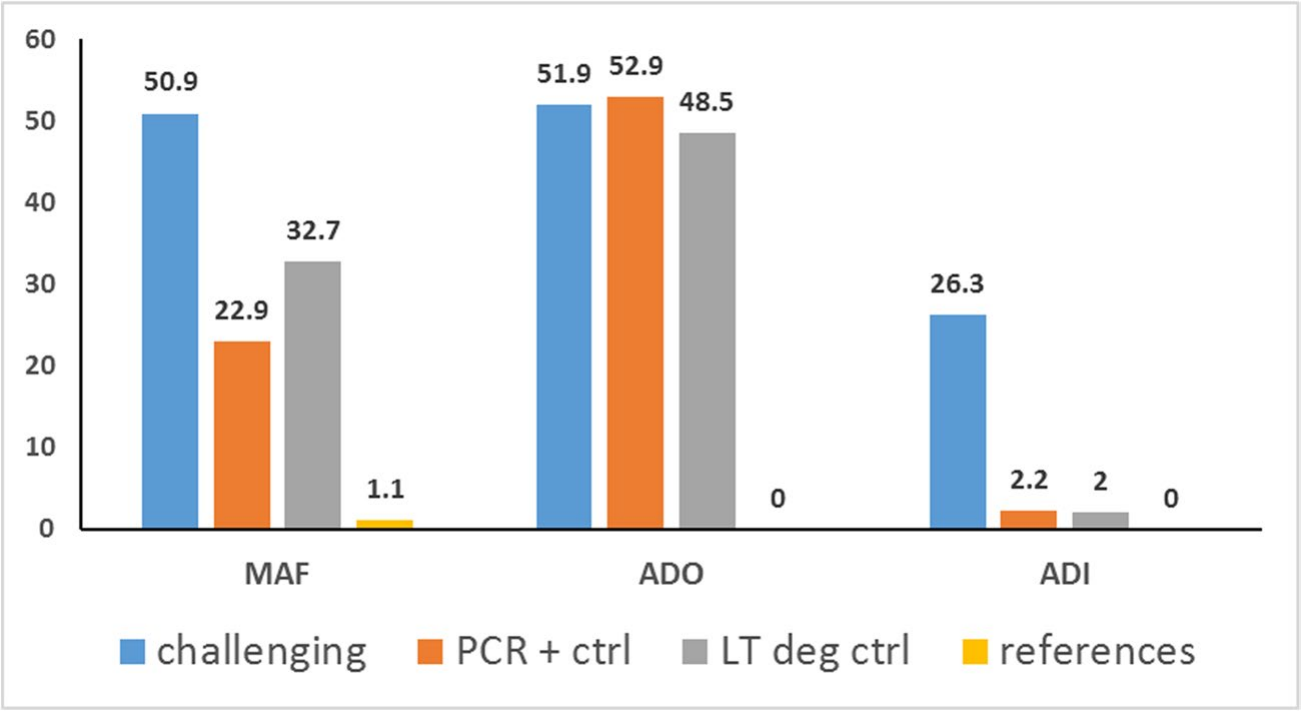
Percentage (*y* axis) of MAF (Major Allele Frequency), allelic drop out (ADO) and allelic drop in (ADI) in autosomal markers. Challenging: challenging samples (n=32); PCR + ctrl: positive PCR control (n=6); LT deg ctrl: low template degraded control (n=9); reference: reference samples (n= 11)

As shown in Fig. 1, 50.9% of the scored markers in the challenging samples showed a MAF flag with higher frequency than positive controls (*p*-value= 0.009). The allelic drop-out (ADO) frequency of challenging samples was quite high (51.9%); however, it was similar to one of positive controls. The allelic drop-in (ADI) frequency was 26.3% in the challenging samples, and therefore higher than the one recorded for the positive controls (*p*-value = 3.1×10^−5^). However, the allelic drop-in frequency showed different values (*p*-value = 0.017) in the three skeletons (32.4% in skeleton A, 11.5% in skeleton B, and 33.5% in skeleton C). In addition, 47% of drop-in events (126 out of 268) changed the original homozygous genotype (e.g. from AA to GG), whereas in the remaining 53% of samples (142 cases) the original homozygous genotype was replaced by a heterozygous genotype (e.g. from CC to CT). Although all samples showed drop-in events, they were mainly clustered in restricted sets of samples, as reported in Table S2 (see also Tables S3-S5, which show the genotyping data of each of the 32 challenging samples).

The origin of the allelic drop-in events in the analysis of SNP markers *via* PCR-MPS has been questioned by numerous researchers [10, 12, 25, 27, 42, 43]. The error rate generated by the Ion Torrent technology is quite high (≥ 1%), but they consisted mainly in insertion/deletion artefacts [44] (whilst the Illumina technology is mainly subjected to misinsertions [45]). Another possibility for drop-in events is the misinsertion occurring during the PCR amplification, an artefact enhanced by DNA degradation through cytosine to uracil transition [46]. The presence of uracil in aged forensic samples has been already described [47], but this mechanism is believed to play a marginal role in the bone samples studied here given the huge number of ADI scored. The most likely explanation is the contamination of the samples. In control samples studied, the allelic drop-in frequency was around 2.1%, what is in agreement with the value of 1.0 to 1.8% found by Turchi et al. in a large set of control and degraded samples [27]. From the same study, however, it emerged that the majority (97.3%) of drop-in events were most likely originated from minimal amounts of exogenous human DNA transferred to the samples rather than artefactual events. Thus, likewise, a contamination issue leading to a DNA mixture should be considered in the case of the challenging samples analysed in the present study.

When analysing STR markers, a DNA mixture has to be considered when more than two alleles per locus are typed [1, 2, 36, 39]. In the case of bi-allelic markers used here, instead, an excess of heterozygosity (Ht) is one of the main findings able to suggest a DNA mixture [12]. In average, the 32 challenging samples showed a heterozygosity of 0.303 with no difference with the positive controls (Ht = 0.252; *p*-value = 0.118). When the genotyping data of challenging samples were used to build the corresponding *consensus* profiles, however, the resulting heterozygosity was 0.785, 0.630, and 0.900 for skeletons A, B, and C respectively. The results for skeletons A and C showed an exceeding heterozygosity degree (*p*-value < 4.7 × 10^−7^) when compared to the expected average value of the Caucasian population (Ht = 0.458) [48]. *Consensus* profiles of each skeleton constructed from the challenging samples are shown in the last column of Table S3, Table S4, and Table S5 for skeletons A, B, and C, respectively. High percentage of drop-in alleles and also the evaluation of the heterozygosity degree of the *consensus* SNP profiles constructed for all three skeletons was of further support for a contamination issue.

### Assessing the identity of the samples

In order to be able to check whether the genotypes achieved from the challenging bone samples match the *reference* genotypes of skeletons A, B, and C, the LRMix and the EuroForMix software were used for LR calculations. The results of the analyses are reported as follows.

#### LRMix analysis of control and reference samples

The LRMix software allows the calculation of the LR under the hypothesis of the same or different contributors for *PH* and *DH* [36, 37]. In this study, we assumed that a single DNA is the contributor of the profile both for *PH* and *DH*. To test the robustness of the LRMix tool on single source DNA sample identification, the SNP genotypes of skeletons A, B, and C, control samples 2800M and FM, and other ten samples from a previous study [27] were used both for intra-sample and inter-sample comparisons. The default setting parameters (ADO probability = 0.10; ADI probability = 0.05) were applied for a total of 225 comparisons. LR values ranging from 4.5 × 10^32^ to 2.7 × 10^36^ were scored in the 15 intra-sample comparisons (see Table S6). In all the remaining cases, e.g. in the 210 inter-samples comparisons, the LR values were < 8.9 × 10^−37^.

Table S2 shows the impact of the ADO and ADI frequencies, as analytical parameters, on the LR values calculated for the 11 samples used to create the *reference* profile for skeletons A, B, and C. For example, the LR of sample #41 increased from 1.73 × 10^34^ (using the default setting parameters) to 6.21 × 10^37^ (using the actual probabilities of ADO and ADI, which corresponded to zero). Altogether, these results confirm that LRMix software is an accurate tool for assessing the identity of single source SNP profiles [36, 37, 49].

#### LRMix analysis of challenging samples

The approach described above was applied to SNP profiles of each challenging sample (see Table S2). In order to state a parameter to consider the minimum number of SNP markers available for a computational analysis, we decided to refer to the number of markers scored for a very low copy number DNA control sample included in our analysis; this sample was library #57 built with 2 pg of 2800M DNA for which only 19 markers could be recorded. Therefore, only challenging bone samples with at least 19 typed markers were considered for the LRMix analysis. By applying this arbitrary threshold, libraries #3 and #5 (with 2 and 17 typed markers, respectively) were discarded. The results of the remaining 30 samples are shown below.

##### LRMix analysis using the default setting parameters

As shown in Table 1, the comparison of the typing results of the 30 challenging samples with the corresponding *reference* profile showed LRd (LR default) values > 1 in eight samples (26.6%), whereas it was < 1 in the remaining 22 samples (73.4%). In addition, as shown in Table 2, in 17 cases (56.6%) the LRd ratios were of “*extremely strong support*” to the *DH* (the challenging sample does not belong to the skeleton from which it was collected). Further computational analyses were performed to explain the reasons of this unexpected result.

**Table 1:** Percentage of samples with LR values > 1 assuming a single DNA source contributor

	Challenging samples (*n*=30)	Positive controls (*n*=15)
LRd	26.6%	86.7%
LRe	46.6%	100%
LRa	70.0%	100%

*LRd* LR using the default setting parameters, *LRe* LR using the ADO frequency as estimated by the LRMix software, *LRa* LR using the ADO and ADI frequencies as calculated sample-to-sample. The positive controls are represented by six PCR positive controls plus nine low template degraded controls

**Table 2:** Assessment of the identity of the samples assuming a single source DNA contributor

LR value	Verbal equivalent	LRd	LRe	LRa
> 1,000,000	*Extremely strong*	7	8	11
1000–1,000,000	*Strong*	0	2	4
2.1–999	*Weak/moderate*	0	4	5
0.5–2.0	*Of no support*	2	2	4
0.49–0.001	*Weak/moderate*	3	2	4
0.0009–0.000001	*Strong*	1	4	1
< 0.000001	*Extremely strong*	17	8	1

The numbers in the last three columns refer to the number of samples (*n* total= 30). *LRd* LR using the default setting parameters, *LRe* LR using the ADO frequency as estimated by the LRMix software, *LRa* LR using the ADO and ADI frequencies as calculated sample-to-sample

##### LRMix analysis using estimated frequencies of allelic drop-out

The calculations were performed by setting the ADO frequencies as estimated by the software using the “*drop out estimation*” option [36]. This option is adopted in the real-case analysis to evaluate the effect of the allelic drop-out, a frequent PCR artefact in forensic samples [1, 2, 36]. Following this approach, 14 samples (46.6%) showed LRe (LR estimated) values > 1 (and therefore supporting the identification of the samples). When the results were pooled, eight samples showed LRe values > 1.0 × 10^6^ (max= 1.1 × 10^18^) leading to an “*extremely strong support*” [38] to the biological identity of the samples (see Table 2). Out of the remaining samples, however, the LRe values supported—with different levels of strength—the *DH* in 14 cases (see Figure S4). The positive control samples (PCR + ctrl plus LT degraded controls) always showed LRe values > 1 (median value = 2.9 × 10^14^; min = 184; max = 1.5 × 10^26^).

##### LRMix analysis using actual frequencies of ADO and ADI

In the third test, the ADO and ADI frequencies were calculated sample-to-sample (see Table S2) and the acquired values were set as analytical parameters in the LRMix software. It is a trivial observation that this approach could not be applied to real casework as the real ADO and ADI frequencies are unknown. Nevertheless, we believe that the observation of the impact of the ADO and ADI with their real frequencies could be of help in explaining the results of the present research work. As shown in Table 1, 21 samples (70.0%) showed LRa (LR actual) values > 1, and therefore supporting the biological identity of the samples (see Table 2). Out of the nine samples with LRa values < 1, three samples showed LRa values of 0.50, 0.60, and 0.90, respectively, and thus providing a neutral support to *PH* or *DH*. Out of the remaining six samples, two samples (C_01 and C_11) showed LRa values of 9.7 × 10^−6^ and 8.5 × 10^−7^, respectively, meaning a “*strong support*” and “*extremely strong support*” to the *DH* (see Figure S4). In conclusion, the genetic profiles obtained from those two samples could be more likely explained if they belong to some other individuals than to the corresponding reference skeleton.

To check if there is a relationship between the LRa values and the frequencies of the allelic drop-ins scored in the samples, the ADI frequencies of the samples with LRa values > 1 were compared with the ADI frequencies of the samples with LRa values < 1. The allelic drop-in frequency was higher in samples with LRa < 1 (0.482 vs. 0.194; *p*-value = 1.7 × 10^−6^; when adding the positive controls, the *p*-value was 1.5 × 10^−7^). The same approach was adopted for comparing the ADO frequencies in the two sets of samples. The allelic drop-out frequency was higher in samples with LRa < 1 (0.608 vs, 0.464; *p*-value = 0.020; when adding the positive controls, the *p*-value was 0.018). In conclusion, these results evidenced that ADI is the main source of error leading to a potential mis-identification of the challenging samples considered in this study.

#### Other analyses with LRMix

To exclude a specimen mislabelling along the experiments as the origin of the mismatch with the corresponding skeleton, further in silico analyses were done on the six partial profiles showing LRa values supporting the *DH* (namely samples A_08, C_01, C_08, C_09, C_10, and C_11). To test this hypothesis, inter-sample comparisons were carried out assuming single source DNA profile. As shown in Table S7, the LR values were always < 1; this result allowed us to reject the hypothesis of mislabelling.

To test the reliability of the results provided by the LRMix tool from partial SNP profiles, the same six samples were compared even with samples 2800M, FM, and other 10 unrelated individuals (see Table S8). For this analysis, the estimated frequency of ADO and the fixed ADI frequency of 0.05 were used; the resulting values ranged from 4.7 × 10^−1^ to 2.0 × 10^−78^ (median value = 1.4 × 10^−18^). Altogether, the results of these additional analyses support the conclusion that partial profiles made of 55 SNP markers (median value) do not create adventitious matches [50].

#### EuroForMix analysis of challenging samples

In order to consider all the information given by the MPS analysis in the evaluation of the profiles obtained from the challenging bone samples and its impact on the LR values in comparison with the semi-quantitative approach, all the challenging bone samples were tested against each corresponding *reference* skeleton with the quantitative continuous model software EuroForMix [37, 39, 49], considering the information given by the number of reads recorded for each scored allele. The hypothesis of a single contributor to the genetic profile was assumed for each comparison, as for the LRMix calculations. The results are reported in Table S9 where they are compared to the LRe values as calculated by the semi-continuous software. The two series of LR values for each challenging sample, belonging to the corresponding three *reference* skeletons, were then plotted as shown in Figure S5. The excellent correlation (*r*^2^ > 0.931) between the results of the two software provide a definitive support to the conclusion that the expected single source profile was achieved in no more than 14 out of 30 samples.

### Y-specific typing

All the eleven samples used as reference samples for the three skeletons yielded results for all 34 Y-SNP markers. In addition, the intra-skeleton comparison showed the same identical alleles. Therefore, three different haplotypes were clearly identified, each one peculiar of a given reference skeleton. The *reference* haplotype of each skeleton is shown in the second column (labelled in red) of Table S10 for skeleton A, Table S11 for skeleton B, and Table S12 for skeleton C.

In agreement with the lower sensitivity of the Y-specific markers of the Precision ID Identity kit compared to the autosomal ones [12, 24, 25, 27], no more than 41.1% of the markers were typed in the challenging samples vs. 45.6% and 61.1% in the PCR + ctrl and the LT degraded control, respectively (see Figures S2-S3). In total, 22 allelic drop-in artefacts (generating spurious haplotypes) were scored in the challenging samples with an average frequency of 5.2%. However, it is noteworthy that these undesirable phenomena did not occur randomly within the samples. In fact, as shown in Tables S2 and S10-S12, up to nine drop-ins were restricted to only two samples (#22 and #32, i.e. the samples with lowest LR values). No drop-in was scored in the 15 positive controls. No read was scored in the seven negative controls. In conclusion, the results of the Y-specific markers analysis are in full agreement with the data of the autosomal identity SNP typing, which was consistent with a contamination issue.

### PCR-CE analysis of the ENCs

As stated above, all ENCs included in the batches of extraction yielded no amplification through 30 cycles of PCR using the Investigator® ESSplex SE QS kit [29]. Since the results of the PCR-MPS suggested a contamination issue for some of the challenging samples, five ENCs were tested through 33 PCR cycles using the PowerPlex ESI 17 Fast kit in the present study. These additional tests were performed to investigate the source of the contamination, and in particular to establish if the contamination issue occurred during the laboratory workflow of the bone samples or if an endogenous human contamination affected the bone samples from the beginning (ab initio).

The results of these analyses showed few alleles with low molecular weights (< 100–120 bp), up to 1.200 rfu in three ENCs (see Figure S6). No amplicon was scored in the NTCs of these PCR-CE-based tests. These findings support the conclusion that human exogenous contamination occurred during the DNA extraction from the bone samples. Nevertheless, since no more than one marker (with two alleles) was scored in each ENC, the comparison with the DNA exclusion database STR profiles was of limited help for identifying the source of contamination through checking the staff’s genetic profiles. However, given the extreme sensitivity of the PCR-MPS, a contamination, even if minimal, could explain the origin of the drop-ins scored in the PCR-MPS analysis of the challenging samples.

## Discussion

Recently, promising results were reported in the STR PCR-MPS typing of Second World War bone samples that had yielded poor results from conventional STR PCR-CE analysis [51]. In this study, we sought to verify whether increased cycles in PCR-MPS would produce useful SNP profiles from aged bone samples which had previously produced no results in conventional STR PCR-CE analyses. To this aim, we tested a set of 32 challenging bone samples by the employment of the Precision ID Identity Panel with 27 cycles of PCR.

Despite that our challenging bone samples had only an average of 6.8 pg of degraded DNA, 30 out of 32 libraries (93.8%) yielded sequencing data for around 63/90 autosomal markers per bone sample. Out of the 30 libraries, 14 (46.7%) yielded single source genetic profiles whose LRe supported—with different levels of strength—the biological identity [38] of the samples (see Table 2 and Figures S4 and S5). However, these excellent results were overshadowed by the problems associated with contamination. In particular, even if 27 cycles of PCR-MPS analysis succeeded in the production of reliable genotyping data from almost half of the extremely challenging samples, it is also true that minute human exogenous contaminations were identified and genotyped in at least 12 cases (40.0%) leading to spurious SNP profiles. Thus, some considerations are needed to address the risk of misleading conclusions in a real-case scenario.

This experiment can be considered a proof-of-concept study; in fact, the genotype of the challenging bone samples was known a priori, a situation which allowed for computational analyses otherwise unfeasible. For instance, ADOs and ADIs can be merely suspected in the real-case analysis, even if the results of duplicate tests are available. Instead, here both ADO and ADI frequencies were available sample-to-sample, which allowed for the assessment of the weight of these artefactual phenomena in the reliability of the genotyping data.

In real-case analyses of SNP markers, a high degree of heterozygosity is one of the main features which can alert the operator about the possibility of human DNA contamination [12, 52]. Instead, here we found that the (expected) high frequencies of ADO were usually balanced by the (unexpected) high frequencies of ADI in challenging samples which led to SNP profiles whose heterozygosity degrees were similar to the ones of the positive controls (*p*-value = 0.118). Nevertheless, it has to be highlighted that the *consensus* profiles given by the challenging samples of skeletons A and C showed exceeding values of heterozygosity (*p*-value < 4.7 × 10^−7^) due to the high number of spurious alleles dropped in the corresponding samples. Similarly, the contamination issue was found even within the Y-specific markers, which therefore show artefactual haplotypes. Thus, altogether, our results highlight the importance of the multi-sample approach to avoid misleading conclusions [1, 2, 41, 42, 46, 53]. In fact, we were only able to suspect this contamination issue by having multiple samples from the same skeleton. It has to be noted, however, that the availability of several samples from the same biological donor is not the rule in the real-case analysis.

Even in routine clinical samples, negative/blank controls are required in order to suspect and/or identify whether the specimens were contaminated by exogenous human DNA throughout the lab workflow [1, 2, 41, 46, 53–55]. The PCR-MPS analysis of the NTCs confirmed the absence of exogenous DNA in the reagents used for the library preparation, which suggested that the contamination issue occurred during upstream procedures [41, 53, 54]. In addition, four ENCs yielded molecular results, even if it was for a very limited number of alleles, thus providing a likely explanation for the high number of allelic drop-ins scored in the challenging samples of skeletons A and C.

From this, the questions emerging now are: what is the source of such contamination, and where does it occur? Exogenous human contamination can occur in any step of the extraction procedure, from the sampling of the bone elements to their pulverisation, etc. [41, 53, 54]. It is important to note that the processing of aged bone samples for genetic analysis is much longer than the processing of any other forensic sample. There are many steps involved in the extraction of DNA from bones and consequently a lot of manual actions conducted by the laboratory staff. Therefore, bones are more prone to exogenous DNA contamination in comparison to other forensic samples. In this work, all extraction procedures were performed in a sterile clean room under a hood with negative pressure dedicated solely to aged bone samples by using UV-radiated reagents and consumable [29, 56]. When isolating DNA from bones, we followed the measures to prevent contamination described previously [56]. In particular, we prevented the surface contamination of bones with exogenous DNA by mechanical and chemical cleaning and UV irradiation. In addition, only the bone samples of skeletons A, B, and C were processed in successive extraction batches included in present study. Therefore, contaminations can be likely ascribed to operator carry-out events even if individual protection devices were always worn. However, minute amounts of exogenous DNA left on the surface of bone cannot be excluded.

The results presented in this paper show that PCR-MPS is very sensitive. PCR-MPS can detect even minute amounts of DNA contamination, especially when the number of PCR cycles is boosted. This is in sharp contrast with the conventional PCR-CE approach, which cannot detect such low levels of DNA contamination. In fact, almost half of the challenging bone samples analysed in this study showed extra-alleles whose origin can be most likely ascribed to a contamination issue. We recommend increasing the number of measures to prevent contamination during excavation, anthropological analysis, and sample storage. People who participate in these work procedures should be strict in their use of protective clothing, masks, and gloves. In addition, the tools used for anthropological measurements should be cleaned sequentially with bleach, water, and ethanol. Also, more rigorous measures could be used to remove surface contamination of the bones, through extending the exposure time of bones to bleach before obtaining the bone powder. This would increase the possibility of more effective degradation of exogenous contamination from the bone surface. For more successful removal of surface contamination, it is also possible to extend the time the bones are exposed to UV radiation. Using both measures (extension of chemical cleaning with bleach and extension of UV irradiation) could reduce or completely eliminate surface contamination due to individuals who were in contact with the bones before the genetic analysis [56]. In addition, it is highly recommended to increase the number of blank/negative controls (in particular the ENCs) which have to be processed alongside the challenging DNA samples, in order to identify the presence of a contamination (for example, one ENC could be included every four samples, as described for archaeological remains [57]). Lastly, to identify the most likely source of this issue, the lab staff working on the bone samples should be typed for the selected PCR-MPS kit. It is however necessary to be aware that all these procedures only help in the identification of contamination but do not protect from its occurrence. Finally, in the cost estimation for a similar routine approach to challenging specimens, it should be considered the rising charge originated from the addition of more blank/negative controls. In this work, part of the ENCs were analysed via PCR-CE with higher number of cycles (33 instead of 30), which represents a cheaper and more flexible tool. Further studies are needed, however, to establish if this could be a sensitive and reliable enough tool in routine analyses.

In conclusion, our study shows that it is possible to perform a PCR-MPS approach by typing identity SNP markers on challenging bone samples which did not provide genetic results using the conventional STR PCR-CE approach. LR values obtained in this study show extremely strong support to the identification of the bone samples. However, we believe that the evaluation of the results should be considered reliable only if a positive identification of the samples is obtained by comparison with a reference sample, through statistical approaches such as RMP or LR. On the contrary, LR values that support the hypothesis of exclusion are inconclusive because it is not possible to exclude that exogenous DNA contamination substantially impacted the genetic results. In fact, the extreme sensitivity of the PCR-MPS [8–10, 42] seems to represent the main limit of its employment on the challenging bone samples analysed in this study.

## Supplementary information

ESM 1 (TIFF 187542 kb)

ESM 2 (XLSX 392 kb)

## Data Availability

The data of this work are available on supplementary material.

## References

[1] McCord BR, Gauthier Q, Cho S, Roig MN, Gibson-Daw GC, Young B, Taglia F, Zapico SC, Mariot RF, Lee SB, Duncan G (2019). Forensic DNA analysis. Anal Chem.

[2] Gill P, Haned H, Bleka O, Hansson O, Dørum G, Egeland T (2015). Genotyping and interpretation of STR-DNA: low-template, mixtures and database matches-twenty years of research and development. Forensic Sci Int Genet.

[3] Butler JM, Coble MD, Vallone PM (2007). STRs vs. SNPs: thoughts on the future of forensic DNA testing. Forensic Sci Med Pathol.

[4] Sobrino B, Brión M, Carracedo A (2005). SNPs in forensic genetics: a review on SNP typing methodologies. Forensic Sci Int.

[5] Budowle B, van Daal A (2008). Forensically relevant SNP classes. Biotechniques.

[6] Alaeddini R, Walsh SJ, Abbas A (2010). Forensic implications of genetic analyses from degraded DNA--a review. Forensic Sci Int Genet.

[7] Butler JM (2022). Recent advances in forensic biology and forensic DNA typing: INTERPOL review 2019-2022. Forensic Sci Int Synerg.

[8] Børsting C, Morling N (2015). Next generation sequencing and its applications in forensic genetics. Forensic Sci Int Genet.

[9] Bruijns B, Tiggelaar R, Gardeniers H (2018). Massively parallel sequencing techniques for forensics: a review. Electrophoresis.

[10] Ballard D, Winkler-Galicki J, Wesoły J (2020). Massive parallel sequencing in forensics: advantages, issues, technicalities, and prospects. Int J Legal Med.

[11] Børsting C, Fordyce SL, Olofsson J, Mogensen HS, Morling N (2014). Evaluation of the Ion Torrent™ HID SNP 169-plex: a SNP typing assay developed for human identification by second generation sequencing. Forensic Sci Int Genet.

[12] Eduardoff M, Santos C, de la Puente M, Gross TE, Fondevila M, Strobl C, Sobrino B, Ballard D, Schneider PM, Carracedo Á, Lareu MV, Parson W, Phillips C (2015). Inter-laboratory evaluation of SNP-based forensic identification by massively parallel sequencing using the Ion PGM™. Forensic Sci Int Genet.

[13] Guo F, Zhou Y, Song H, Zhao J, Shen H, Zhao B, Liu F, Jiang X (2016). Next generation sequencing of SNPs using the HID-Ion AmpliSeq™ Identity Panel on the Ion Torrent PGM™ platform. Forensic Sci Int Genet.

[14] Buchard A, Kampmann ML, Poulsen L, Børsting C, Morling N (2016). ISO 17025 validation of a next-generation sequencing assay for relationship testing. Electrophoresis.

[15] Meiklejohn KA, Robertson JM (2017). Evaluation of the Precision ID Identity Panel for the Ion Torrent™ PGM™ sequencer. Forensic Sci Int Genet.

[16] Huang E, Liu C, Zheng J, Han X, Du W, Huang Y, Li C, Wang X, Tong D, Ou X, Sun H, Zeng Z, Liu C (2018). Genome-wide screen for universal individual identification SNPs based on the HapMap and 1000 Genomes databases. Sci Rep.

[17] Li R, Zhang C, Li H, Wu R, Li H, Tang Z, Zhen C, Ge J, Peng D, Wang Y, Chen H, Sun H (2017). SNP typing using the HID-Ion AmpliSeq™ Identity Panel in a southern Chinese population. Int J Legal Med.

[18] Miyashita K, Ochiai E, Tamura T, Osawa M (2015). Kinship Analysis Based on SNP Data from the HID-Ion AmpliSeqTM Identity Panel. J ForensicInvestigation.

[19] van der Heijden S, de Oliveira SJ, Kampmann ML, Børsting C, Morling N (2017). Comparison of manual and automated AmpliSeq™ workflows in the typing of a Somali population with the Precision ID Identity Panel. Forensic Sci Int Genet.

[20] Liu J, Wang Z, He G, Zhao X, Wang M, Luo T, Li C, Hou Y (2018). Massively parallel sequencing of 124 SNPs included in the precision ID identity panel in three East Asian minority ethnicities. Forensic Sci Int Genet.

[21] Kampmann ML, Buchard A, Børsting C, Morling N (2016). High-throughput sequencing of forensic genetic samples using punches of FTA cards with buccal swabs. Biotechniques.

[22] Sun L, Fu L, Liu Q, Zhou J, Ma C, Cong B, Li S (2019). Population data using Precision ID Identity Panel in a Chinese Han population from Hebei Province. Forensic Sci Int Genet.

[23] Christiansen SL, Jakobsen B, Børsting C, Udengaard H, Buchard A, Kampmann ML, Grøndahl ML, Morling N (2019). Non-invasive prenatal paternity testing using a standard forensic genetic massively parallel sequencing assay for amplification of human identification SNPs. Int J Legal Med.

[24] Gettings KB, Kiesler KM, Vallone PM (2015). Performance of a next generation sequencing SNP assay on degraded DNA. Forensic Sci Int Genet.

[25] Salata E, Agostino A, Ciuna I, Wootton S, Ripani L, Berti A (2016). Revealing the challenges of low template DNA analysis with the prototype Ion AmpliSeq™ Identity panel v2.3 on the PGM™ Sequencer. Forensic Sci Int Genet.

[26] Avila E, Cavalheiro CP, Felkl AB, Graebin P, Kahmann A, Alho CS (2019). Brazilian forensic casework analysis through MPS applications: statistical weight-of-evidence and biological nature of criminal samples as an influence factor in quality metrics. Forensic Sci Int.

[27] Turchi C, Previderè C, Bini C, Carnevali E, Grignani P, Manfredi A, Melchionda F, Onofri V, Pelotti S, Robino C, Sorçaburu-Ciglieri S, Tagliabracci A, Fattorini P (2020). Assessment of the Precision ID Identity Panel kit on challenging forensic samples. Forensic Sci Int Genet.

[28] Tiedge TM, Nagachar N, Wendt FR, Lakhtakia A, Roy R (2021). High-throughput DNA sequencing of environmentally insulted latent fingerprints after visualization with nanoscale columnar-thin-film technique. Sci Justice.

[29] Zupanc T, Podovšovnik E, Obal M, Zupanič Pajnič I (2021). High DNA yield from metatarsal and metacarpal bones from Slovenian Second World War skeletal remains. Forensic Sci Int Genet.

[30] Ewing MM, Thompson JM, McLaren RS, Purpero VM, Thomas KJ, Dobrowski PA, DeGroot GA, Romsos EL, Storts DR (2016). Human DNA quantification and sample quality assessment: developmental validation of the PowerQuant(®) system. Forensic Sci Int Genet.

[31] Zupanič Pajnič I, Previderè C, Zupanc T, Zanon M, Fattorini P (2022). Isometric artifacts from polymerase chain reaction-massively parallel sequencing analysis of short tandem repeat loci: an emerging issue from a new technology?. Electrophoresis.

[32] Thermo Fisher Scientific (2019). Identity Thermo Fisher Scientific, Precision ID SNP Panel with the HID Ion S5TM/HID Ion Gene StudioTM S5 System, Application Guide, MAN0017767.

[33] Thermo Fisher Scientific (2018) Torrent Suite™ Software 5.10, User Guide, MAN0017598, Carlsbad, CA

[34] Hussing C, Kampmann ML, Mogensen HS, Børsting C, Morling N (2018). Quantification of massively parallel sequencing libraries - a comparative study of eight methods. Sci Rep.

[35] Thermo Fisher Scientific (2017). Converge software.

[36] Gill P, Haned H (2013). A new methodological framework to interpret complex DNA profiles using likelihood ratios. Forensic Sci Int Genet.

[37] Bleka Ø, Eduardoff M, Santos C, Phillips C, Parson W, Gill P (2017). Open source software EuroForMix can be used to analyse complex SNP mixtures. Forensic Sci Int Genet.

[38] European Network of Forensic Science Institute (2015). ENFSI guideline for evaluative reporting in forensic science.

[39] Bleka Ø, Storvik G, Gill P (2016). EuroForMix: an open source software based on a continuous model to evaluate STR DNA profiles from a mixture of contributors with artefacts. Forensic Sci Int Genet.

[40] Qiagen (2021) Investigator® ESSplex SE QS Handbook, HB-1963-003, Hilden

[41] Poinar H (2003). The top 10 list: criteria of authenticity for DNA from ancient and forensic samples. Int. Congr. Ser..

[42] Hofreiter M, Sneberger J, Pospisek M, Vanek D (2021). Progress in forensic bone DNA analysis: lessons learned from ancient DNA. Forensic Sci Int Genet.

[43] Zupanič Pajnič I, Zupanc T, Leskovar T, Črešnar M, Fattorini P (2022). Eye and hair color prediction of ancient and Second World War skeletal remains using a forensic PCR-MPS Approach. Genes (Basel).

[44] Bragg LM, Stone G, Butler MK, Hugenholtz P, Tyson GW (2013). Shining a light on dark sequencing: characterising errors in Ion Torrent PGM data. PLoS Comput Biol.

[45] Schirmer M, Ijaz UZ, D’Amore R, Hall N, Sloan WT, Quince C (2015). Insight into biases and sequencing errors for amplicon sequencing with the Illumina MiSeq platform. Nucleic Acids Res.

[46] Dabney J, Meyer M, Pääbo S (2013). Ancient DNA damage. Cold Spring Harb Perspect Biol.

[47] Fattorini P, Marrubini G, Ricci U, Gerin F, Grignani P, Cigliero SS, Xamin A, Edalucci E, La Marca G, Previderé C (2009). Estimating the integrity of aged DNA samples by CE. Electrophoresis.

[48] https://www.ncbi.nlm.nih.gov/snp/. Accessed 11 January 2022

[49] Costa C, Figueiredo C, Amorim A, Costa S, Ferreira PM, Pinto N (2022). Quantification of forensic genetic evidence: comparison of results obtained by qualitative and quantitative software for real casework samples. Forensic Sci Int Genet.

[50] Yousefi S, Abbassi-Daloii T, Kraaijenbrink T, Vermaat M, Mei H, van’t Hof P, van Iterson M, Zhernakova DV, Claringbould A, Franke L, LM t H, Slieker RC, van der Heijden A, de Knijff P, BIOS consortium, t Hoen PAC (2018). A SNP panel for identification of DNA and RNA specimens. BMC Genomics.

[51] Zupanič Pajnič I, Fattorini P (2021). Strategy for STR typing of bones from the Second World War combining CE and NGS technology: a pilot study. Forensic Sci Int Genet.

[52] Peyrégne S, Prüfer K (2020). Present-day DNA contamination in ancient DNA datasets. Bioessays.

[53] Pääbo S, Poinar H, Serre D, Jaenicke-Despres V, Hebler J, Rohland N, Kuch M, Krause J, Vigilant L, Hofreiter M (2004). Genetic analyses from ancient DNA. Annu Rev Genet.

[54] Rohland N, Hofreiter M (2007). Ancient DNA extraction from bones and teeth. Nat Protoc.

[55] Sehn JK, Spencer DH, Pfeifer JD, Bredemeyer AJ, Cottrell CE, Abel HJ, Duncavage EJ (2015). Occult specimen contamination in routine clinical next-generation sequencing testing. Am J Clin Pathol.

[56] Pajnič IZ (2016). Extraction of DNA from human skeletal material. Methods Mol Biol.

[57] Thomas MTP, Rudbeck L, Willerslev E, Hansen AJ, Smith C, Penkman KEH, Prangenberg K, Nielsen-Marsh CM, Jans ME, Arthur P, Lynnerup N, Turner-Walker G, Biddle M, Kjølbye-Biddle B, Collins MJ (2004). Biochemical and physical correlates of DNA contamination in archaeological human bones and teeth excavated at Matera, Italy. J Archaeol Scie.

